# Empirical Analysis of Safe Distance Calculation by the Stereoscopic Capturing and Processing of Images Through the Tailigator System

**DOI:** 10.3390/s19225044

**Published:** 2019-11-19

**Authors:** Gerd Christian Krizek, Rene Hausleitner, Laura Böhme, Cristina Olaverri-Monreal

**Affiliations:** 1 Department Applied Mathematics and Physics, University of Applied Sciences Technikum Wien, 1200 Vienna, Austria; hausleit@technikum-wien.at (R.H.); boehmelaura93@gmail.com (L.B.); 2 Department Sustainable Transport Logistics 4.0, Johannes Kepler University, 4040 Linz, Austria

**Keywords:** tailgating, safety distance, image processing, stereoscopy

## Abstract

Driver disregard for the minimum safety distance increases the probability of rear-end collisions. In order to contribute to active safety on the road, we propose in this work a low-cost Forward Collision Warning system that captures and processes images. Using cameras located in the rear section of a leading vehicle, this system serves the purpose of discouraging tailgating behavior from the vehicle driving behind. We perform in this paper the pertinent field tests to assess system performance, focusing on the calculated distance from the processing of images and the error margins in a straight line, as well as in a curve. Based on the evaluation results, the current version of the Tailigator can be used at speeds up to 50 km per hour without any restrictions. The measurements showed similar characteristics both on the straight line and in the curve. At close distances, between 3 and 5 m, the values deviated from the real value. At average distances, around 10 to 15 m, the Tailigator achieved the best results. From distances higher than 20 m, the deviations increased steadily with the distance. We contribute to the state of the art with an innovative low-cost system to identify tailgating behavior and raise awareness, which works independently of the rear vehicle’s communication capabilities or equipment.

## 1. Introduction

Advanced Driver Assistant Systems (ADAS) that focus on maintaining a safe distance from a leading vehicle, such as Forward-Collision Warning (FCW), use a combination of cameras, radar, and laser to detect whether the distance between vehicles is safe and alert the driver when that is not the case.

The Adaptive Cruise Control (ACC) ADAS uses radar or cameras to detect traffic and automatically adjust the vehicle’s speed to maintain a safe distance from the leading vehicle by applying longitudinal control algorithms to the throttle and/or the brake [1]. Cooperative ADAS (coADAS) that are based on Vehicle-to-Vehicle (V2V) communication, such as the cooperative ACC, have the ability to measure the velocity of other vehicles very precisely and determine if a vehicle ahead has decelerated. Even a penetration rate of 40% of vehicles with V2V communication capabilities suffices to prevent accidents in scenarios with rear-end collisions [2].

Even given that ACC systems have some restrictions related to their response to stopped or slowed traffic if the vehicle was driving at a high speed, or that they require driver input in some traffic conditions below a certain speed threshold, it remains the case that vehicles with ADAS are much more accurate than humans. They not only help to prevent accidents, but they also have been proven beneficial to the environment and traffic flow.

It has been argued that almost every automobile company is manufacturing vehicles with ACC systems on board that enable automatic vehicle following in the longitudinal direction [3]. While automakers are making efforts to include ADAS features in more than just their premium cars, these systems are being offered as optional equipment in many economy and low-priced models. For example, according to a Bosch evaluation based on the 2014 registration statistics, only 8% of the nearly three million registered passenger cars in Germany were equipped with ACC systems [4].

**Figure 1:**
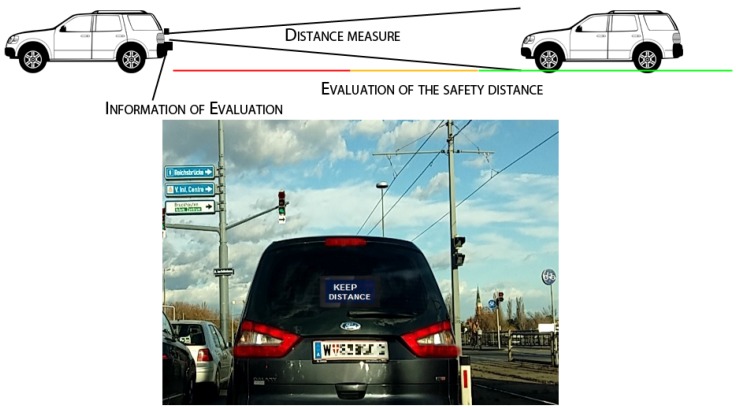
Tailigator Advanced Driver Assistant Systems (ADAS) in which visual data related to the safety distance is provided to the rear vehicle in real-time [6].

In order to contribute to active safety in the road and prevent or mitigate road crashes, we propose in this work a low-cost FCW system that gathers information through the stereoscopic capturing and processing of images by rear cameras. They are located in the rear part of the leading vehicle, with the purpose of discouraging tailgating behavior from the vehicle driving behind through a message (see Figure 1).

It has been argued that fluency of communication between users and systems is determined by the kind of information provided to the driver [5]. Adhering to this statement, our Tailigator system seeks to reduce road fatalities and damage by using traffic condition warnings in real-time to alert drivers when the longitudinal safety distance between two vehicles is not maintained and poses a danger to both [6,7]. This kind of visual information might improve safety by making drivers more mindful with regard to potential tailgating behavior, as it emphasizes the interpersonal collaborative nature of driving in order to improve road safety, rather than just safety through sensor-equipped vehicles.

As an extension of the work presented in [8], in which a detailed error analysis of the distance calculation proposed in our solution was provided based on several measurement procedures and roadway geometry, we implement in this work a new software-architecture approach. We rely on the use of multithreading via 3rd party libraries as QT to investigate if the time difference between the stereoscopic cameras of 0.2 s that was due to the limitations of the previous implementation approach can be improved by integrating real-time capabilities in the system.

Our contribution consists of a novel visualization of messages that is independent of the communication capabilities of the following vehicle, being thus applicable for example in scenarios with low penetration rates of connected and autonomous vehicles [8].

## 2. Related Work

ADAS, such as ACC, are able to adjust the vehicle’s speed after having acquired information from a forward-looking radar that is installed in the front part of a vehicle [9]. This approach is in stark contrast to our proposed system, which uses information that has been collected by camera sensors mounted in the rear part of the leading vehicle. This location of the sensors allows us to aim at improving driving behavior in situations in which the leading vehicle is forced to increase speed and disregard speed limits as an effect of aggressive tailgating behavior from a following vehicle.

A lot of research in the context of object detection for rear-end collision avoidance systems has been performed in recent years. One example is the combining of images from several cameras to increase driver visual perception, accomplished through the mapping of two simultaneous images using parameters, such as camera shot angle, camera focal length, and the virtual square of the area of interest [10].

In the same line of research, a comprehensive analysis of the mathematical models used to measure distances based on stereoscopic pictures was provided in [11]. Detection of tailgating behavior has been investigated in several other works, for example through algorithm frameworks based on video-based data in urban road junctions [12]. Approaches to discourage tailgating can include feedback systems, such as the ones described in [13,14]. In these works, the authors assessed the impact of several headway feedback systems on behavior change based on visual and auditory cues. In addition, by using individual vehicle records and a multivariate linear regression data analysis to research the effect of certain messages on the amount of close and aggressive driving, the authors in [15] detected significant differences in behavior, depending on the messages used to characterize an unsafe distance to a leading vehicle.

In [16], the authors coped with challenges regarding video analysis for detecting tailgating behavior, such as variations of background and pose uncertainty, by an improved Gaussian Mixture Model (IGMM) for background. They also combined a Deterministic Nonmodel-Based approach with Gaussian Mixture Shadow Model (GMSM) to remove shadows, in the end establishing a tracking strategy and computing the similarity of color histograms. A different approach was presented in [17], where optical stereoscopy was used in a stationary environment in which a 3D camera was used to provide the two images for processing. The authors concluded that even if the camera did not meet the required accuracy settings, results from a phase-only-correlation method were successful.

An implementation comparable to our presented approach is the rear-end collision warning system from [18], which also uses the rear view camera of the vehicle. The vehicle’s surroundings are analyzed based on measurements of distance, speed and acceleration, all relative to the following vehicle. A warning is issued as soon as the approaching vehicle enters a non-safe distance. The main idea behind this system was to prevent accidents caused by inattentive drivers. Experiments from off-line video tests were proven to be successful, the system having a potential application in warning the driver of the leading vehicle.

In the same line of research but focusing on eliciting a behavior change from the driver in the following/rear vehicle, in [6,8], the stereoscopic capturing and processing of images by rear cameras was used to calculate in real time the distance between a leading and a following vehicle.

If a certain threshold value regarding the distance between both vehicles was exceeded, the leading vehicle displayed a message in the rear part reminding tailgaters to be rational in the event that the tailgating was intentional on the part of the rear driver.

Additionally, relying on the communication between the two vehicles, an in-vehicle system was compared to the developed rear-mounted distance warning system under lab-controlled conditions in terms of their impact on driver response in [14]. Results showed that both systems influenced the driver in keeping a time gap of two seconds.

We contribute in this work to the state of the art and extend the technological approach of the last three referenced papers by integrating real-time capabilities in the system. We then assess its performance in a field test as described in the next sections.

## 3. System Implementation

The original system described in [6] was based on a Raspberry Pi 1 Model B+ and designed and implemented from scratch aiming at a stable approach with a real-time performance. There were several shortcomings (e.g., 0.2 s time difference between cameras, iteration duration between 3 and 6 s, and unstable frame capturing) that needed to be tackled in order for the system to be applicable on the road. An improved version of the approach was described in [8], in which a detailed error analysis of the distance calculation was provided based on a measurement procedure and roadway geometry.

**Figure 2:**
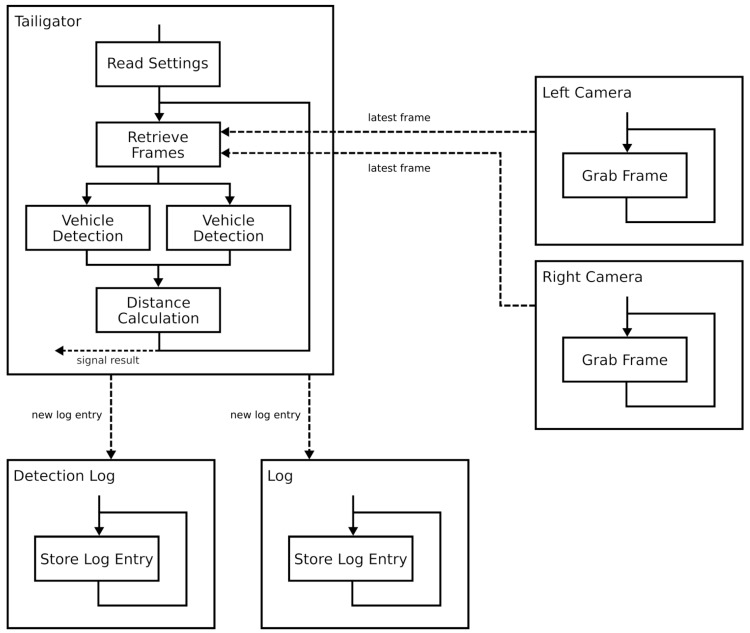
Software architecture of the improved Tailigator.

In the work presented in this paper, we upgraded the original Raspberry Pi 1 Model B+ to a Raspberry Pi 3 Model B board using Logitech C270 web cameras. The upgrade was possible due to the multi-threaded design of the system that made it well suited for modern multi-core processors as the one integrated in the Raspberry Pi 3. The software was written in the C++ programming language using the QT framework with its signal/slot mechanism and the OpenCV 2.4 library. A graphical overview of the software architecture can be seen in Figure 2.

As it is depicted in the figure, the Tailigator software reads in the settings from a file that is written in JSON format. Resolution, frame rate, log directory path, and camera device paths are some of the data that can be found in the settings file. The next step is the retrieving of the latest frames from the cameras. Each camera is handled by a single thread that has continuous access to frames and that stores the last one that is located in the memory for the Tailigator thread so that it can be retrieved when necessary. When the frames are retrieved, two vehicle detection threads are created and each one is transferred to a frame. These two threads detect the vehicles in parallel, thus effectively reducing the detection time in half. After the detection, the distance is calculated and signaled to a listening user. Then, the Tailigator thread starts retrieving the latest pair of frames over again. With this approach, we managed to increase the resolution from 640 × 480 to 864 × 480 and the frame rate from 5 fps to 15 fps, while also reducing the time difference between the cameras from 0.2 s to 0.067 s. Furthermore, the iteration duration was reduced to approximately 0.8 s.

## 4. Field Test Evaluation of the System

To analyze the Tailigator system performance against the predicted error models, we performed an empirical evaluation of the following parameters:

- The errors pertained to measurements in curved road sections.
- The distance measurement on a straight road section and its accuracy.

The distance to an object $D_{object}$ placed in front of the cameras was measured following the method presented in [8] and using the following parameters in Equation (1) by [11]: the distance between the cameras $D_{cameras}$, the horizontal field of view $\varphi_{0}$, the horizontal pixel resolution (pixel number) $Px_{h}$, and the horizontal pixel difference to the same object in both pictures in pixels $Px_{L}-Px_{R}$.

(1) $$D_{object}=\frac{D_{cameras}*Px_{h}}{2*\tan\left(\frac{\varphi_{0}}{2}\right)*\left(Px_{L}-Px_{R}\right)}.$$

### 4.1. Curved Road Sections

**Figure 3:**
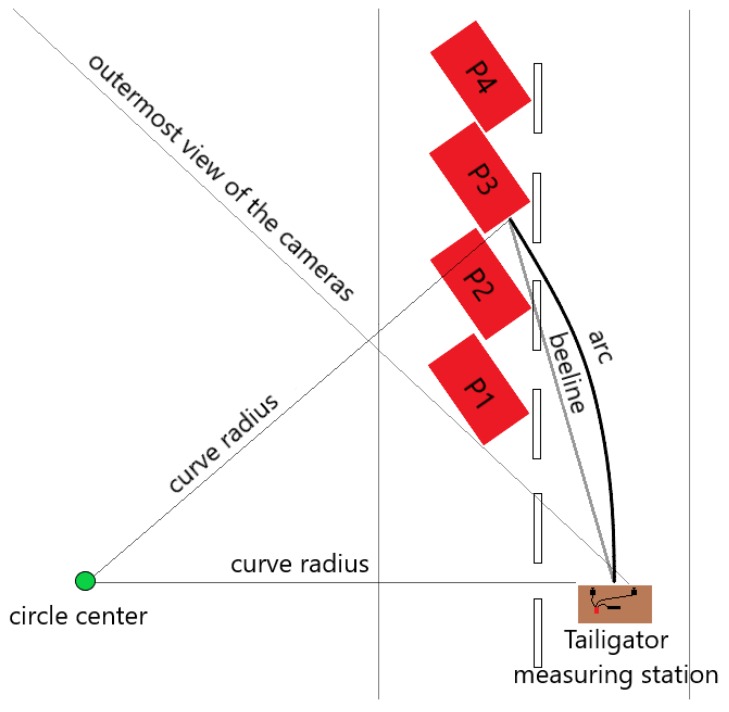
Graphic depicting the set up to measure with the four different car positions.

For the measurements in curved road sections, the rear vehicle was placed in a way that the right edge of its bumper could still be detected by both cameras. This resulted in four curve radii with which the measurements were carried out. A graphical overview of the measurement setup in the curve is provided by Figure 3.

As explained in [8], the effective driving distance between the leading and following vehicles in circular curves of radius R is denoted by Equation (2), where the angle α is calculated as shown in Equation (3) producing a negative value. The deviation between the distance of the circle’s segment and the measured distance by the system is given by $Dev_{D}=D_{curveobject}-D_{object}$, where the deviation is denoted by Equation (4).

(2) $$D_{curveobject}=R\cdot \alpha ,$$

(3) $$\alpha =\arctan\left(\frac{D_{object}}{\sqrt{R^{2}-D_{object}^{2}}}\right),$$

(4) $$DEV_{D}=R\left|\arctan\left(\frac{D_{object}}{\sqrt{R^{2}-D_{object}^{2}}}\right)\right|-D_{object}.$$

### 4.2. Straight Road Section

To perform the measurements on a straight road section, the realdistance range between the leading and the rear vehicle was selected within a range of 3 to 30 m. To test the independence of the car model used, we performed the experiments with two different vehicles that were named Testcar1 and Testcar2. At every position of the realdistance between both vehicles, several measurements were performed.

The image recognition procedure of the Tailigator aimed at detecting the vehicles in the two parallel recorded camera frames. If a recognition of the vehicle in both camera frames was not possible at the same time, the calculation of the distance failed and a event was logged. If a vehicle was detected by the Tailigator in both camera frames, the distance was calculated and recorded. The distancedeviation was analyzed as a result of the difference between the distanceobject (denoted by $D_{object}$) and the realdistance.

## 5. Evaluation Results

### 5.1. Distance on Curved Road Sections

The results of the measurements in the curved road sections in Table 1 showed a similar course of increasing measurement errors with increasing distances.

**Figure 4:**
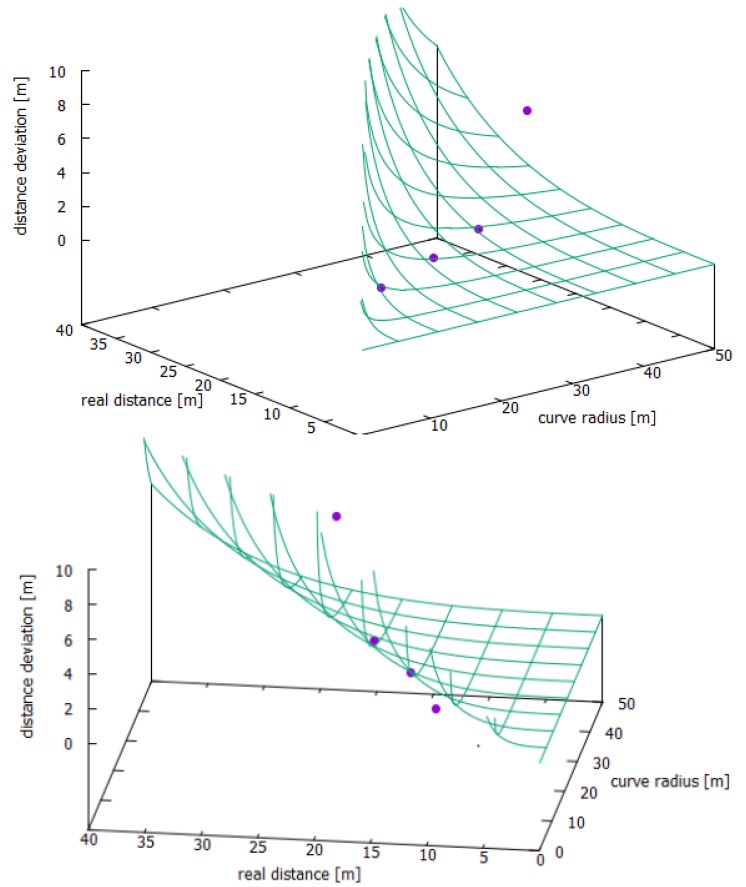
Average values of the measurements (purple dots) embedded in the theory error curve (green net).

This can be seen in a graphical way by the theory error curve provided by [6] in Figure 4. As shown in the graph, the mean values of the measured distances of the four positions are consistent with the calculated theoretical error curve, even though the error bars do not reach the real distance curve.

The measurements showed similar characteristics both on the straight line and in the curve. At close distances, between 3 and 5 m, the values deviated to a higher extent from the real value. At average distances, around 10 to 15 m, the Tailigator achieved the best results. From distances more than 20 m, the deviations increased steadily with the distance.

### 5.2. Distance on Straight Road Sections

**Figure 5:**
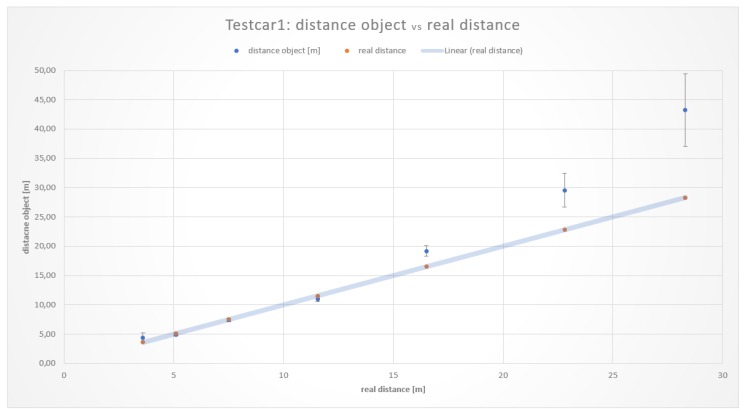
Comparison of the realdistance with the measured $\bar{distance\;object}$.

**Figure 6:**
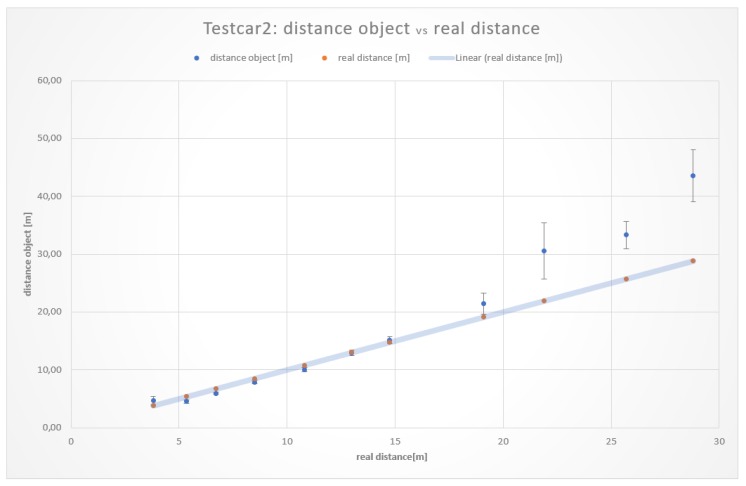
Comparison of the realdistance with the measured $\bar{distance\;object}$.

The measurements resulting from the distance on a straight road section of both vehicles Testcar1 and Testcar2 are illustrated in Table 2 and Table 3, respectively. Graphical results showing the comparison of the real distance with the measured distanceobject are depicted in Figure 5 and Figure 6.

As it can be seen, the averages at shorter distances were closer to the real values than those at longer distances. This behavior is not surprising. Results concerning the different vehicles used in the experiments showed that the measurement values for distances in the range of 5 to 15 m were the most accurate and consistent with each other. For distances more than 15 m, the measurement error increased significantly.

The measurement approach to determine the safe distance calculation without errors by the stereoscopic capturing and processing of images through the Tailigator system showed accurate results in a range of approximately 5 to 16 m. Measurements less than 5 m were made, in order to determine the exact lower bounds of the working range.

**Figure 7:**
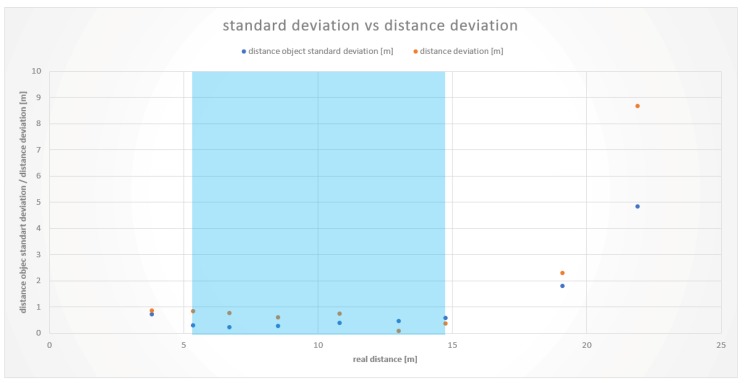
Working area of the Tailigator on a straight road section (blue marked area).

The respective measurements at 3.6 and 3.8 m showed deviations from the real values to a higher extent and problems in vehicle identification. The lower limit of the working range of the Tailigator is therefore established in the range of 3.8 to 5.1 m. Figure 7 illustrates the working area of the Tailigator on a straight road section (blue marked area).

**Figure 8:**
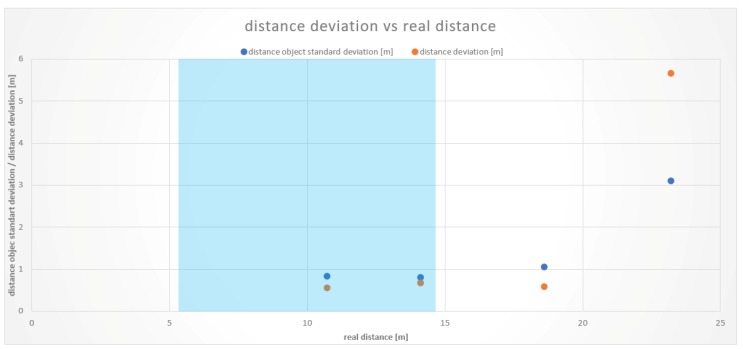
Working area of the Tailigator on curved road sections (blue marked area).

At the upper bound of the working range, there were comparable results between measurements on a straight line and in a curve. At the first test vehicle, a distance of 16.5 m resulted in an average distance deviation of 2.7 m. The measurements with the second test vehicle showed smaller but consistent distance deviations. At a distance of 14.7 m, there was an average deviation of 0.36 m and, at 19.1 m, a mean difference of 2.3 m. These values and the resulting working area of the second test vehicle are depicted in Figure 8.

**Figure 9:**
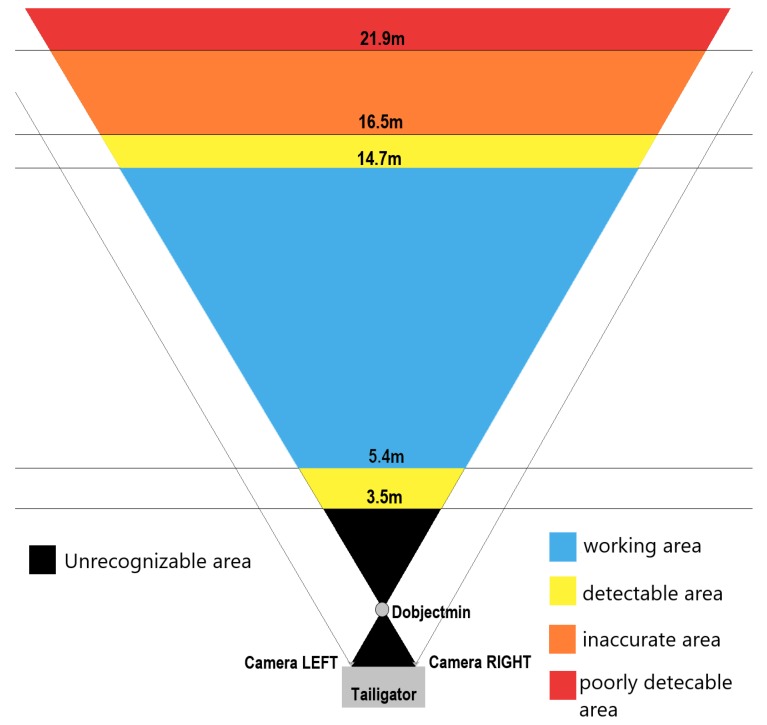
Working areas of the Tailigator related to the distance.

Results from the measurements in the curved road sections showed a high distance deviation from a distance of 23.2 m on. The reason for this is that the rear vehicle could no longer be detected clearly by the cameras. A significantly higher vehicle recognition rate occurred less than 18.8 m, determining the working area within this range in straight and curved road sections. The boundaries of the Tailigator are shown graphically in Figure 9.

The working area of the Tailigator is related to the speed-dependent critical safety distance. The blue area in Table 4 identifies the working area of the Tailigator in this context.

## 6. System Redesign on the Basis of the Resulting Evaluation

Based on the results from the empirical evaluation, we increased the frame rate to 30 fps, the iterations per second, and the resolution of the frames. This was achieved by using an optimized version of the OpenCV 3.2 library and by replacing the Raspberry Pi with the UP board.

The UP Board is the same size as the Raspberry Pi 3 Model B and is also equipped with the same 40-pin header, but it provides double the processing power of the Raspberry and three independent USB 2.0 ports, while the ports on the Raspberry are shell shared. These independent USB ports are especially important for a stable frame rate of 30 fps at higher resolutions. For practical purposes, it should be noted that the UP board costs nearly three times as much as the Raspberry Pi 3 Model B.

The OpenCV 3.2 library was compiled with architecture-dependent optimization in order to get more performance out of the processors. For the Raspberry we used the option -mfpu=neon-fp-armv8 and for the UP Board the options -msse, -msse2, -msse3, -mssse3, -msse4.1, and -msse4.2.

Since minimizing the time difference of the cameras was a requirement, only the results for 30 fps are presented. At lower frame rates the iterations per second (IPS) were slightly higher (about 10% for 15 fps). The Raspberry Pi 3 can be used for resolutions up to 864 × 480. If more than that, the USB bandwidth is insufficient, resulting in slowdowns or corrupted images, or both.

The independent USB ports of the UP Board, on the other hand, allow the use of much higher resolutions. Therefore, the UP Board was able to process an iteration in less than a second at a resolution of 1280 × 960. Table 5 summarizes the results of the tests at 30 fps.

Summarizing the results, a reduction of the time difference from 0.067 s to 0.034 s could be achieved, while also significantly reducing the iteration duration from 0.8 s to less than 0.27 s (UP Board)/0.56 s (RPi3) at 864 × 480.

## 7. Conclusions and Future Work

The empirical analysis presented in this work supports the theoretical error model presented in [8]. Relevant system modifications have been conducted to achieve a low-cost rear-end collision assistant, while improving the time and optical resolution of the system.

Based on the evaluation results, the current version of the Tailigator can be used at speeds up to 50 km per hour without any restrictions, since the critical safety distance resides fully within the working range. At more than 50 km per hour, the working range of the Tailigator would be exceeded. To cover the entire range of permitted speeds in Austria with the critical safety distance in the working area of the Tailigator, it would be necessary that the Tailigator provide a working range of up to 55 m. However, the purpose of the Tailigator system is to inform and alert the driver of the rear vehicle of their tailgating behavior, therefore, such a long range is not required and the visibility of the tailgating warning sign to rear drivers (see Figure 1) in this regime is questionable.

Future work will include the analysis of the transfer potential to the thermal infrared spectrum in order to extend its use to any road condition and scenario, as well as the implementation of a systematic error model to assess the system that resulted from the evaluation presented in this work.

## Figures and Tables

**Table 1:** Measurement results on the curved road.

RealDist.BeeLine[m]	$\bar{\mathrm{Distance}\;\mathrm{Object}}\;\left[m\right]$	$\sigma_{\mathrm{Distance}\mathrm{Object}}\left[m\right]$	Dist.Deviation[m]	UsableData	Dataw.Error
23.2	28.87555556	3.115907216	5.675555556	9	216
18.6	19.19456989	1.054359843	0.594569892	186	190
14.1	14.77720588	0.815804707	0.677205882	136	136
10.7	11.2602193	0.833063158	0.560219298	228	241

**Table 2:** Measurement results of the test vehicle Testcar1.

RealDistance[m]	$\bar{\mathrm{Distance}\;\mathrm{Object}}\;\left[m\right]$	$\sigma_{\mathrm{Distance}\mathrm{Object}}\;\left[m\right]$	DistanceDeviation[m]	UsableData	DatawithError
3.60	4.37	0.81	0.77	24	85
5.10	4.90	0.18	0.19	78	78
7.50	7.37	0.28	0.12	86	86
11.55	10.97	0.42	0.57	93	93
16.50	19.20	0.87	2.70	110	110
22.80	29.57	2.86	6.77	127	129
28.30	43.24	6.20	14.94	397	422

**Table 3:** Measurement results of the test vehicle Testcar2.

RealDistance[m]	$\bar{\mathrm{Distance}\;\mathrm{Object}}\left[m\right]$	$\sigma_{\mathrm{Distance}\mathrm{Object}}\;\left[m\right]$	DistanceDeviation[m]	UsableData	DatawithError
3.80	4.65	0.71	0.85	18	184
5.35	4.51	0.29	0.83	298	299
6.70	5.94	0.21	0.75	194	194
8.50	7.89	0.27	0.60	205	205
10.80	10.04	0.37	0.75	141	141
13.00	12.90	0.45	0.09	389	389
14.75	15.11	0.58	0.36	128	128
19.10	21.40	1.80	2.30	199	200
21.90	30.56	4.82	8.66	66	119
25.70	33.31	2.35	7.61	132	133
28.80	43.55	4.49	14.75	87	87

**Table 4:**
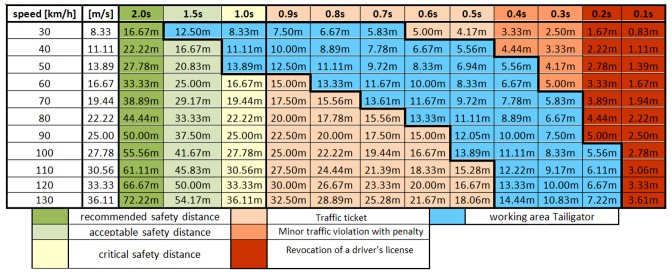
Distance (meters) related to time (seconds) and speed (kilometers per hour).

**Table 5:** IPS at 30 fps.

30 fps	Iterations per Second (IPS)				
	640 × 480	864 × 480	1024 × 576	1280 × 720	1280 × 960
RPi3	2.91	1.79			
UP	5.83	3.80	2.49	1.40	1.04
